# Mediation effects of depression and anxiety on social support and quality of life among caregivers of persons with severe burns injury

**DOI:** 10.1186/s13104-019-4761-7

**Published:** 2019-11-27

**Authors:** Kevin Bonsu, Nuworza Kugbey, Martin Amogre Ayanore, Ethel Akpene Atefoe

**Affiliations:** 1 Clinical Psychology Unit, 37 Military Hospital, Accra, Ghana; 2 School of Public Health, University of Health and Allied Sciences, Ho, Volta Region Ghana; 3 School of Medicine, University of Health and Allied Sciences, Ho, Volta Region Ghana

**Keywords:** Caregiving, Depression, Anxiety, Social support, Quality of life, Ghana

## Abstract

**Objective:**

Caregiving is associated with several psychosocial challenges including stress, depression and anxiety. These challenges have been found to have significant negative impacts on the health and wellbeing of caregivers, but the mechanisms of these effects are poorly understood. This study examined whether depression and anxiety serve as mediators between social support and quality of life caregivers of persons with severe burns injury.

**Results:**

A sample of 100 caregivers of persons with severe burns injury were administered questionnaires to assess their depression, anxiety, social support and quality of life. Findings show that depression and anxiety were negatively correlated with quality of life whereas social support was positively correlated with quality of life. Results further showed that only depression significantly mediated the link between social support and quality of life among the caregivers. These findings emphasize the need to screen caregivers for common mental health problems and provide them support in the caregiving process to promote their health and wellbeing.

## Introduction

Burns injuries have become an important public health challenge in low and middle income countries including Ghana due to increasing numbers and associated challenges for victims and their relations [1, 2]. The nature of the treatment requires efforts from several professionals and non-professionals such as family caregivers. Caregivers play an integral role in treatment and recovery of persons with chronic medical conditions. Individuals with severe burn injuries require support from both formal and informal caregivers as informal healthcare support is seen as an important aspect of the treatment process [3]. The supports provided by informal caregivers to individuals come in the form of financial, social, emotional and spiritual [4, 5]. However, the provision of such supports by caregivers are not without consequences to their own health and wellbeing as the demands of caregiving can be overwhelming [6, 7]. Consequently, caregivers’ quality of life may be significantly impacted.

Several factors have been found to be associated with quality of life among caregivers of persons with chronic medical conditions including depression, anxiety and caregiver burden [7, 8]. However, one of the key resources in times of stress is social support. Social support may be actual or perceived but its positive impacts on health and wellbeing outcomes of caregivers of varied medical conditions have been reported in the literature [9, 10]. Social support has been associated with decreased stress, depression and anxiety levels among caregivers [11, 12]. In the same vein, caregiver stress, depression and anxiety contribute significantly to decreased quality of life among caregivers [13, 14].

Despite several studies in the literature demonstrating the individual contributions of depression, anxiety and social support to quality of life among caregivers, the mechanisms underlying these relationships have received little attention. For instance, limited studies have reported a sense of defeat and entrapment as mechanisms underlying the negative impact of depression and anxiety on short and long term health outcomes including quality of life [15–17]. Pertinent literature within the global and Ghanaian contexts on quality of life among adult caregivers of persons with severe burns injury are lacking and therefore, this study examined the direct and indirect influences of social support on quality of life through depression and anxiety, which are known risk factors for decreased quality of life.

## Main text

### Methods

#### Design and procedure

A cross-sectional study design was employed, and 100 participants representing a response rate of 91.7% were administered questionnaires that measured the study variables. Ethical clearance was obtained from the Institutional Review Board of the Noguchi Memorial Institute for Medical Research, University of Ghana, Legon (NMIMR-IRB CPN 095/12-13). Informed consent was obtained from all the participants prior to their involvement in the study. The questionnaires were interviewer-administered at the Burns unit of a Teaching hospital in Ghana.

#### Measures

A set of questions were developed to measure demographic characteristics such as age, sex, duration of care, sex and age of burns patients. *Quality of life* was measured with the World Health Organization Quality of Life Assessment—Bref (WHO 1996). The scale consists of 26 questions developed to provide a quality of life measure that would be applicable cross-culturally (WHO 1996). Responses to the items on the scale range from 1 to 5. The composite quality of life score was used in this study with higher scores indicating better overall quality of life. The scale had internal consistency values of the four main domains of functioning ranging between .81 and .92 in this study. *Depression* was measured with the Beck Depression Inventory [18]. The Beck Depression Inventory is a self-report measure of depression and includes 21 items measuring cognitive, affective, behavioural, interpersonal, and somatic aspects of depression. It was developed as an indicator of depressive symptomatology and severity. Higher scores are indicative of more depressive symptoms. The scale had an internal consistency value of .93 in this study. *Anxiety* was measured with the Beck Anxiety Inventory [19]. This scale consists of 21 items which measure the severity of anxiety in adults and adolescents. Each item was scored 0, 1, 2 or 3, and total anxiety score was computed with higher scores denoting an increasing severity of anxiety symptoms. The scale had an internal consistency value of .89 in this study. *Social support* was measured with the multidimensional scale of perceived social support [20]. This scale measures perceived social support from three sources: significant others, family and friends. It consists of 12 items rated on a 7-point Likert scale with scores ranging from ‘very strongly disagree’ (1) to ‘very strongly agree’ (7). A total social support score was calculated for each participant with higher scores indicating higher perceived social support. The scale had an internal consistency value of .88 in this study.

#### Data analysis

Data analyses were performed with the use of SPSS version 22.00 and PROCESS Macro [21]. Bivariate correlation analyses were conducted to determine the relationships among the study variables. Mediational analysis was conducted using PROCESS [21] to determine the direct and indirect effects of social support on quality of life through of depression and anxiety. Duration of caregiving role, age of participants and age of the burns patients were included in the analysis as control variables as they correlated significantly with quality of life in the preliminary analysis. A bootstrap method using iterations of computed samples (10,000) recommended by [21] was used to determine the significance of the indirect effects. Unlike other methods for testing mediations, the bootstrap method does not violate assumptions of normality and is therefore recommended for small sample sizes [21]. All analysis were conducted at the .05 alpha level (*p* < .05).

### Results

#### Demographic profiles of the participants

For the caregivers sampled, the majority (79%) were females with only 21% being males. The caregivers had an average age of 33.20 years with a standard deviation of 8.88 years. The average years of caregiving role was 2.80 years with a standard deviation 3.27 years. It was also observed that the majority (62%) of the caregivers were providing care for their other relatives (40%) and spouses (22%). Almost half (49%) of the caregivers reported being married, 39% reported being single and the remaining 12% reported either being divorced or widowed. For the patients’ characteristics, a mean age of 36.23 years with a standard deviation of 17.81 years were reported. A little over half of the burns patients (53%) were females.

#### Descriptive statistics

The descriptive statistics of the study variables are summarized in Table 1. As can be seen, data on all the study variables were normally distributed with skewness and kurtosis values within the acceptable ranges of ± 2 and ± 7 respectively [22].

**Table 1:** Summary of descriptive statistics of the study variables

Variables	Minimum	Maximum	Mean	SD	Skewness	Kurtosis
Social support	18.00	77.00	2.80	3.27	.32	−.51
Depression	.00	57.00	33.20	8.88	.22	−1.18
Anxiety	.00	49.00	36.23	17.81	.50	−.06
Quality of life	32.00	124.00	45.63	13.17	−.19	−.25

#### Bivariate correlations among the study variables

Results from the Pearson correlations showed that social support was significantly and negatively correlated with levels of depression (*r* = −.55, *p *< .001) and anxiety (*r* = −.46, *p* < .001) but positively correlated with overall quality of life (*r *= .58, *p* < .001) among the participants. As can be seen in Table 2, depression (*r* = −.84, *p* < .001) and anxiety (*r* = −.50, *p *< .001) were significantly and negatively correlated with overall quality of life among the participants.

**Table 2:** Correlation matrix of the relationships among the study variables

Variables	1	2	3	4	5	6	7
1. Duration in years	1						
2. Age (participants)	.44***	1					
3. Age (patients)	−.06	.20*	1				
4. Social support	.27**	−.16	.02	1			
5. Depression	−.25*	.27**	.10	−.55***	1		
6. Anxiety	−.12	.23*	.05	−.46***	.61***	1	
7. Quality of life	.27**	−.29**	−.24*	.58***	−.84***	−.50***	1

*p < .05; **p < .01; ***p < .001

#### Depression and anxiety as mediators of the link between social support and quality of life

**Fig. 1:**
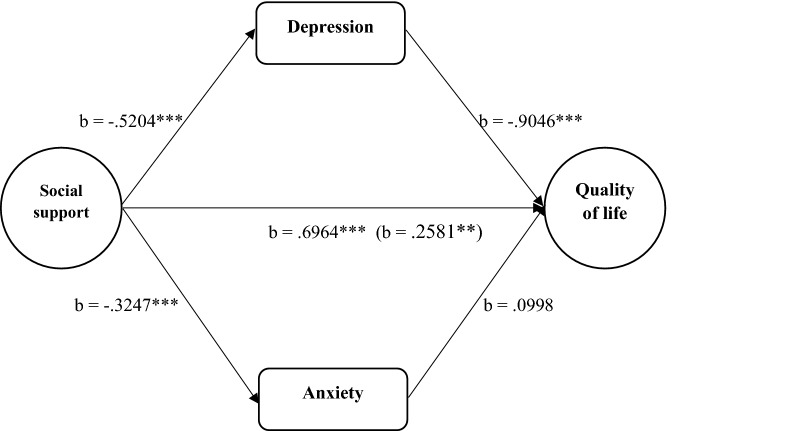
Summary of the direct and indirect effects of social support on quality of life through depression and anxiety

As can been seen in Fig. 1, social support predicted decreased depression (*b* = −.5204, *t *= − 4.8917, *p* < .001) and anxiety (*b* = −.3247, *t* = − 3.9869, *p* < .001) levels among the caregivers. Whereas depression predicted decreased quality of life (*b* = −.9046, *t *= − 9.9319, *p* < .001), anxiety did not significantly predict quality of life (*b* = .0998, *t* = .8392, *p *= .4035) among the caregivers. A bootstrap confidence interval for the indirect effect of social support on quality of life through depression (*b *= .4707) was entirely above zero (.2737 to .6702). However, social support did not have any significant indirect effect on quality of life through anxiety (*b* = −.0324) as the bootstrap confidence interval included zero (−.1207 to .0419). There was evidence of significant direct effect of social support on overall quality of life among the caregivers (*b *= .2581, *t *= 2.6908, *p *< .01).

### Discussion

Findings showed that depression and anxiety were significantly and negatively correlated with quality of life among caregivers of persons with severe burns injury. That is, higher levels of depressive and anxiety symptoms among the caregivers are associated with decreased quality of life. The significant negative relationships between quality of life and both depression and anxiety among caregivers have been reported in the literature [13, 14]. Depression and anxiety are known risk factors for poor quality of life among varied populations including caregivers as the symptoms of depression and anxiety constitute distress which affect an individual’s perception of subjective wellbeing [7, 13, 23]. For instance, depression is characterized by experiences of sadness, appetite problems, sleep problems, loss of energy and a sense of hopelessness. These may result from the burden of proving care for a loved one [7]. These experiences may affect caregiver functioning as they might not have the skills and resources to adapt to their caregiving roles. Social support was also found be significantly and positively correlated with quality of life among caregivers of persons with severe burns injury. Social support plays a significant role in adjusting to stressful situations such as caregiving. Caregiving places significant economic, social and emotional burden on caregivers and availability of social support whether perceived or received could mitigate the negative impact of caregiving on the quality of life of caregivers. For burns patients, caregivers are intimately involved in the treatment and recovery process, thus, lack of support might negatively influence their health and wellbeing. This significant positive relationship between social support and quality of life is consistent with the “buffering” hypothesis of social support in stressful situations [24] and other empirical studies on social support among caregivers [23, 25].

The indirect influences of social support on quality of life among the caregivers showed depression to be a significant mediator in the relationship between social support and quality of life. That is, increased social supported was significantly associated with increased quality of life through decreased depression but not anxiety. Social support predicted decreased depression level which is consistent with what has been reported in the literature [11, 12] and decreased depression predicted improved quality of life. Thus, decreased depression serves as one of the possible mechanisms underlying the positive impact of social support quality of life among caregivers.

These findings are significant in the face of scant literature on quality of life among caregivers of burns injury as the findings imply that appropriate and cost-effective psychosocial interventions aimed at supporting caregivers to deal with the burdens of their caring role should be prioritized in the healthcare delivery process. Another implication of the findings is that there is the need for routine screening of caregivers for possible mental health problems and how they cope with their burden of caregiving to inform appropriate interventions as part of the holistic healthcare delivery for patients with burns injury. The implication of the findings for research is that coping strategies employed by caregivers and how these strategies affect their health and wellbeing outcomes should be examined. However, it is important to note that the sample size for this study was relatively small and this could affect the extent to which these findings can be generalized. Although burns injuries are on the rise with their associated problems, identification of caregivers becomes difficult as the caregiving is mostly shared by family members, friends and significant others. Thus, future research should employ larger samples which would include all persons involved in the caregiving process.

### Conclusion

Based on the findings from this study, it is concluded that caregivers of persons with burns injury experience common mental health problems which affect their quality of life. However, availability of social support serve to improve quality of life of caregivers of persons with burns injury through reduction in common mental health problems especially depression which needs to be the focus on psychosocial interventions.

## Limitations

The study was conducted at only one tertiary health facility and the findings may not be generalizable to the general Ghanaian caregiving population. In addition, the cross-sectional survey does not allow for causal inferences as such results should be interpreted with such limitations in mind. Despite these shortcomings, this is one of the first studies on caregivers of severe burns patients in Ghana and provides the basis for further research into this area.

## Data Availability

The datasets used and/or analysed during the current study are available from the lead and corresponding authors on reasonable request.

## Abbreviations

CI: confidence interval
SPSS: Statistical Package For Social Sciences
WHO: World Health Organization
